# Supplementary material for: Development of Lightweight and High-Performance Ballistic Helmet Based on Poly(Benzoxazine-co-Urethane) Matrix Reinforced with Aramid Fabric and Multi-Walled Carbon Nanotubes
Source: Polymers (Basel). 2020 Dec 3;12(12):2897. doi: 10.3390/polym12122897 (PMC7761712; doi:10.3390/polym12122897)
Supplement: Supplementary file 1 [file polymers-12-02897-s001.pdf]

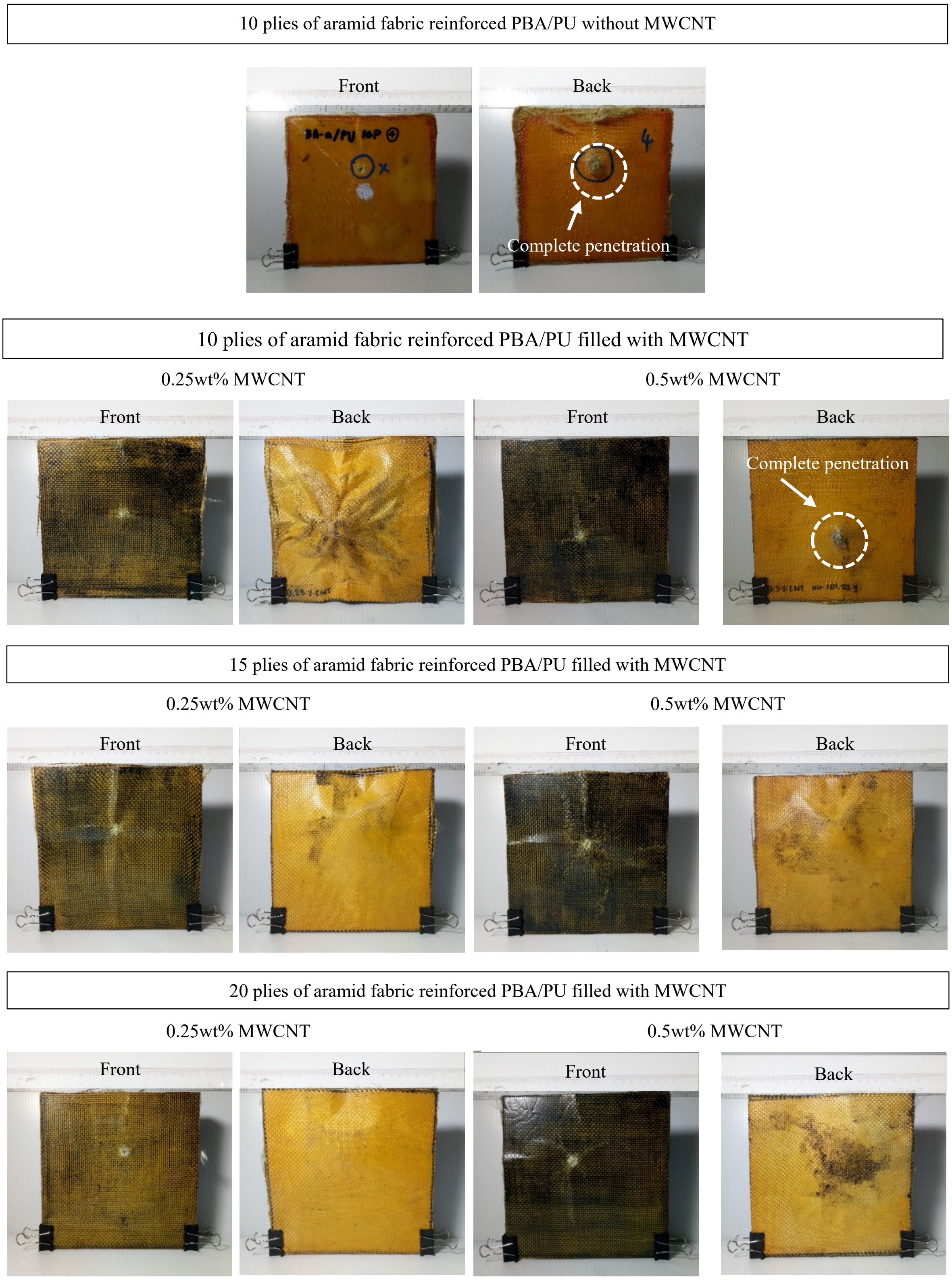
10 plies of aramid fabric reinforced PBA/PU without MWCNT
Front
Back
Complete penetration
10 plies of aramid fabric reinforced PBA/PU filled with MWCNT
0.25wt% MWCNT
0.5wt% MWCNT
Front
Back
Front
Back
Complete penetration
15 plies of aramid fabric reinforced PBA/PU filled with MWCNT
0.25wt% MWCNT
0.5wt% MWCNT
Front
Back
Front
Back
20 plies of aramid fabric reinforced PBA/PU filled with MWCNT
0.25wt% MWCNT
0.5wt% MWCNT
Front
Back
Front
Back

Figure S1: Ballistic impact of aramid fabric reinforced PBA/PU filled MWCNT specimens tested at level II according to NIJ-STD-0106.0
